# Application of Mendelian randomization to assess host gene–gut microbiota correlations in patients with esophageal cancer

**DOI:** 10.3389/fmicb.2023.1309596

**Published:** 2023-12-21

**Authors:** Zhenhu Zhang, Guodong Zhang, Zhulan Huang, Yamin Shi, Dong Wang

**Affiliations:** ^1^Department of Thoracic Surgery, Shandong Provincial Hospital Affiliated to Shandong First Medical University, Jinan, China; ^2^Department of Ultrasound Medicine, Longgang District Maternity and Child Healthcare Hospital of Shenzhen City, Shenzhen, Guangdong, China; ^3^Department of Foreign Languages, Shandong University of Finance and Economics, Jinan, China

**Keywords:** esophageal cancer, gut microbiota, Mendelian randomization, single nucleotide polymorphism, meta-analysis

## Abstract

**Background:**

Increasing evidence suggests that esophageal cancer (ESCA) may be correlated with gut flora. However, their causal connection remains unclear. This study aimed to evaluate potential causal linkages and gene–gut microbiome associations between the gut microbiota and ESCA using Mendelian randomization (MR).

**Methods:**

We analyzed the data using genome-wide association studies. The exposure factor and outcome variable were the gut microbiota and ESCA, respectively. The MR-Egger method, weighted median, inverse-variance weighted method, heterogeneity test, sensitivity analysis, and multiplicity analysis were used for the MR analysis. And it was validated using an external dataset. Further meta-analysis was performed to validate the robustness of this relationship. Finally, we annotated single nucleotide polymorphisms in the gut microbiota that were causally associated with ESCA to explore possible host gene-gut microbiota correlations in patients with ESCA.

**Results:**

We identified four species with potential associations with ESCA. Three of these species had a negative causal relationship with ESCA (odds ratio (OR): 0.961; 95% confidence interval (CI): 0.923–0.971; *p* = 0.047 for *Romboutsia*; OR: 0.972; 95% CI: 0.921–0.961; *p* = 0.018 for *Lachnospira*; OR: 0.948; 95% CI: 0.912–0.970; *p* = 0.032 for *Eubacterium*). A positive causal relationship was observed between one bacterial group and ESCA (OR: 1.105; 95% CI: 1.010–1.072; *p* = 0.018 for *Veillonella*). External datasets show the same trend. This is further supported by meta-analysis. None of the data showed pleiotropy, and leave-one-out analysis indicated the reliability of these findings. The gut microbiomes of patients with ESCA may correlate with the 19 identified genes.

**Conclusion:**

Our data indicate a potential causal link between these four gut bacteria and ESCA and identify a correlation between host genes and gut microbiota in ESCA, offering novel therapeutic options.

## Background

Esophageal cancer (ESCA) is one of the most common cancers globally, with the sixth highest mortality rate according to global cancer data (Sung et al., 2021). Surgery is an effective treatment for ESCA, but in advanced ESCA, the 5-year survival rate of patients remains less than 25% even after surgery (Oppedijk et al., 2014). Chemotherapy is commonly used as a treatment for ESCA but has unavoidable side effects such as toxicity and drug resistance (He et al., 2021). Additionally, epidemiological data indicates that the incidence of ESCA is increasing annually, gravely endangering human health (Uhlenhopp et al., 2020). Therefore, identifying factors potentially associated with ESCA can provide an essential basis for the early prevention of ESCA.

Increasing evidence has shown that the gut microbiota and ESCA are closely related (Cheung et al., 2022; Muszynski et al., 2022; Ohkusa et al., 2022; Baba et al., 2023; Sugimoto et al., 2023). Further, significant variations have been reported in the composition and abundance of fecal microorganisms between patients with ESCA and healthy controls. Notably, these differences are closely correlated with the severity of the disease, suggesting that the gut microbiota may play a significant role in the development of ESCA (Li et al., 2022; Lin et al., 2022). Moreover, gut microbiota can alter genome-wide methylation levels in ESCA, which may be one of the mechanisms influencing the malignant behavior of ESCA cells (Baba et al., 2023). Exposure to antibiotics leads to changes in the gut microbiota, which in turn increase the risk of developing ESCA; the risk of developing the disease increases with the duration of antibiotic exposure (Thanawala et al., 2023). Therefore, investigating the potential link between the gut microbiota and disease to prevent and treat ESCA is crucial.

Although current research reveals that the gut microbiota and ESCA are related, the results are susceptible to confounding factors. Mendelian randomization (MR) is a genetic technique frequently used to investigate causal links between exposures and outcomes and prevents confounding variables in common observational studies because genetic variants are randomly assigned at conception (Smith and Ebrahim, 2003).

We investigated the potential causative relationship between gut microbiota and ESCA using MR to provide a proper theoretical foundation for understanding the interaction between ESCA and gut microbiota. We further identified genes related to the single nucleotide polymorphisms (SNPs) in the gut microbiota obtained by MR analysis. Our results may help identify novel therapeutic options for ESCA treatment.

## Methods

### Research methods

**Figure 1:**
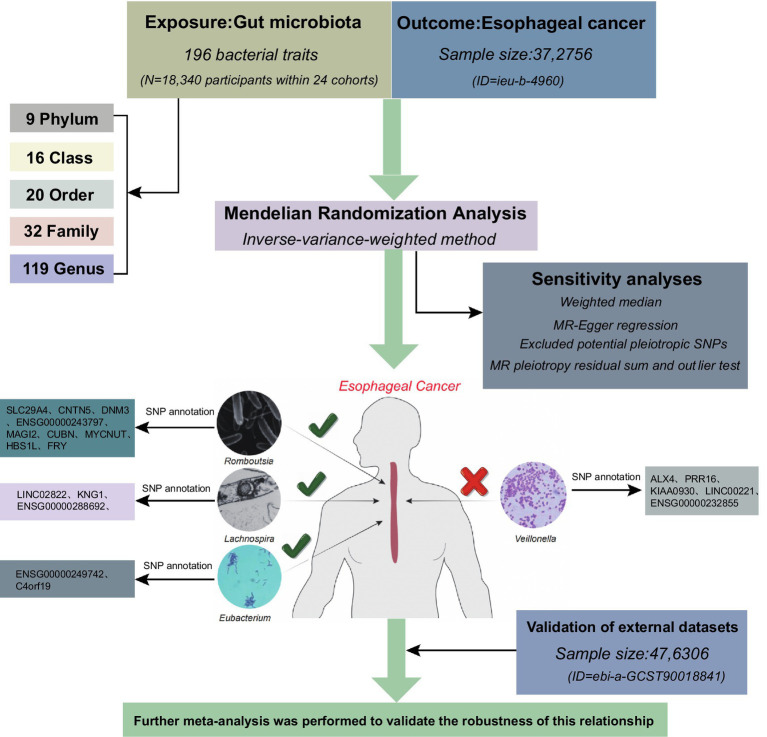
The workflow of the present study.

In this study, heterogeneity, sensitivity, and multiplicity analyses were conducted in addition to MR analyses using genome-wide association studies (GWAS) information to evaluate the causal relationship between gut microbiota and ESCA. MR studies must satisfy three core assumptions of association, independence, and exclusivity: (i) the selected SNPs should be significantly associated with the exposure (intestinal microbiota); (ii) the SNPs must be independent of potential confounders between the exposure and the outcome; and (iii) there is no direct relationship between the SNPs and the outcome (ESCA), and the causal linkage can only be made through the intestinal flora. The workflow is illustrated in Figure 1.

### Data sources

Gut microbiota data from the most recent GWAS meta-analysis, comprising 24 cohorts and 18,340 participants, were used in this investigation (Kurilshikov et al., 2021). ESCA data for the experimental and validation groups were obtained from the UK Biospecimen Repository,^1^ the experimental group included 372,756 samples and the validation group included 476,306 samples (Table 1).

**Table 1:** Details of the genome-wide association studies and datasets used in our analyses.

Exposure or outcome	Sample size	Ancestry	Links for data download	PMID
Human gut microbiome	18,340 participants	Mixed	https://mibiogen.gcc.rug.nl	33462485
Esophageal cancer (Training Group)	3,72,756 participants	European	https://gwas.mrcieu.ac.uk/datasets/ieu-b-4960/	31516927
Esophageal cancer (Validation Group)	4,76,206 participants	European	https://www.ebi.ac.uk/gwas/studies/GCST90018841	34594039

### Instrument selection

A total of 196 bacterial traits, including 9 phyla, 16 classes, 20 orders, 32 families, and 119 genera, were retained after initially removing 15 bacterial qualities without specific names. SNPs significantly related to the gut microbiota were chosen at the genome-wide level (*p* < 1.0 × 10^−5^, R^2^ < 0.001, and clumping distance = 10,000 kb) to fulfill the first MR hypothesis that SNPs must be strongly associated with the gut microbiota. Second, to ensure that the genetic variants were not associated with potential confounders (smoking, heavy alcohol consumption, hot beverages, pickles), a query was performed in the Phenoscanner database.^2^ This step was performed to ensure that the SNPs were not associated with known confounders and, ultimately, to obtain SNPs significantly associated with the gut microbiota, serving as instrumental variables. Next, we calculated the proportion of variance (R^2^). We calculated the F-statistic using the following formula: R = 2 × MAF × (1-MAF) × β^2^, F = R^2^ (n-k-1)/K (1-R^2^), where “MAF” is the minor allele frequency, “N” denotes the exposed GWAS sample size, and “K” is the number of SNPs. *F* > 10 confirmed the absence of a mild instrumental variable bias. The process was completed by annotating the SNPs using an internet database.^3^

### Statistical analysis

The weighted median approach, MR-Egger method, and random effects inverse variance weighting (IVW) method were used in the MR analysis. Statistical significance was set at *p* < 0.05. The IVW approach was primarily employed to analyze these studies (Burgess and Thompson, 2017), and methods such as MR-Egger and weighted median were used to complement the IVW method (Verbanck et al., 2018). Additionally, we applied Cochran’s Q method to evaluate heterogeneity among SNPs. We performed several heterogeneity tests, including the MR-Egger intercept test and a sensitivity analysis, to ensure the robustness of our results. The leave-one-out test was used for the sensitivity analysis to determine outliers among the final SNPs. All data were analyzed using the R packages “Two-Sample-MR” and “MR-PRESSO” in R software (version 4.3.0).

## Results

### Main results of the 196 bacterial traits with the risk of ESCA

**Figure 2:**
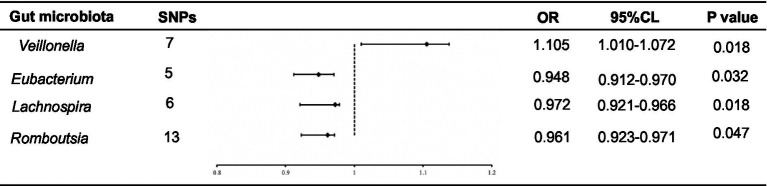
Forest plot of the associations between genetically determined 4 bacterial traits with the ESCA.

The F-statistics for the 196 bacterial characteristics, ranging from 21.63 to 144.84 and with mean values exceeding 10, suggested a robust connection with exposure. We screened for SNPs strongly associated with the gut microbiota (*p* < 1.0 × 10^−5^), and the linkage disequilibrium parameter was set (R^2^ < 0.001, kb = 10,000). Thereafter, the data of the final ESCA were extracted from GWAS, and 196 intestinal flora were merged with the final ESCA sequentially. SNPs with palindromic sequences were excluded from the analysis. Finally, we obtained four gut microbiota samples with potential associations with ESCA using the IVW method (Figure 2).

Using the IVW method, we found that *Romboutsia* was negatively associated with ESCA (odds ratio (OR): 0.961; *p* = 0.047). After screening for F-statistics and excluding chain imbalances, 13 SNPs related to ESCA were included (Supplementary Table S1). In the weighted median approach, the results for the association between *Romboutsia* and ESCA remained stable (*p* = 0.02). To evaluate the stability of these findings, we performed the MR-Egger test on the loci of the included SNPs. No possible horizontal pleiotropy was found (*p* = 0.96), indicating that instrumental variables did not significantly alter the outcomes through mechanisms other than exposure. Cochran’s Q test results showed no significant heterogeneity among the SNPs (*p* = 0.45).

Similarly, *Lachnospira* was negatively associated with ESCA (OR = 0.972; *p* = 0.018). After screening, six SNPs associated with ESCA were included (Supplementary Table S2). In the weighted median approach, the genus *Lachnospira* was weakly associated with ESCA (*p* = 0.08). The SNPs were then subjected to an MR-Egger test, which revealed no apparent level of multiple effects (*p* = 0.96). According to Cochran’s Q analysis (*p* = 0.45), no discernible heterogeneity was observed among the selected SNPs. Additionally, using the IVW method, we discovered that the genus *Eubacterium* was adversely linked with ESCA (OR: 0.948; *p* = 0.032). Overall, five SNPs associated with ESCA were included (Supplementary Table S3). Again, no pleiotropy (*p* = 0.60) or heterogeneity (*p* = 0.79) was observed. In contrast, using the IVW method, we found that *Veillonella* was positively associated with ESCA (OR: 1.105; *p* = 0.018). The seven SNPs associated with ESCA identified after screening (Supplementary Table S4) were free of pleiotropy (*p* = 0.87) and heterogeneity (*p* = 0.41).

**Figure 3:**
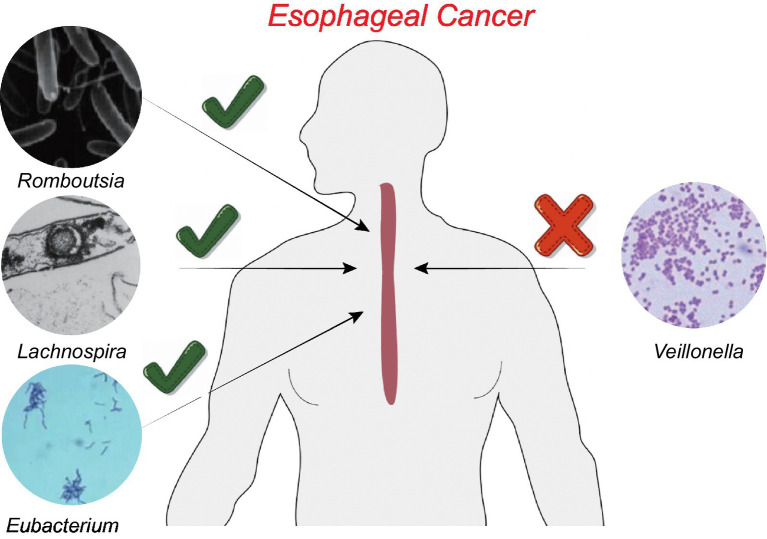
Patterns of the relationship between four gut microbiota and ESCA.

We conducted a “leave-one-out” sensitivity analysis to confirm the impact of each SNP on the overall causation. The findings revealed that none of the SNPs exhibited significant differences when one SNP was excluded (Supplementary Figure S1). Finally, the results were plotted using a pattern map (Figure 3). Using an online database, we identified 19 genes that may be associated with the gut microbiota of patients with ESCA (Table 2).

**Table 2:** SNP annotation of intestinal flora IVs.

		id	chr	Start	End	Strand	Gene_ids	Gene_names
Genus	Romboutsia	rs10279978	7	5279756	5279756	+	ENSG00000164638	SLC29A4
		rs11221428	11	99794319	99794319	+	ENSG00000149972	CNTN5
		rs16843578	1	171934745	171934745	+	ENSG00000197959	DNM3
		rs28603357	7	106766378	106766378	+	ENSG00000243797	ENSG00000243797
		rs34302036	7	78507408	78507408	+	ENSG00000187391	MAGI2
		rs61841503	10	16977560	16977560	+	ENSG00000107611	CUBN
		rs62504452		−1	−1			
		rs7109293		−1	−1			
		rs75200530		−1	−1			
		rs75987356	2	15926583	15926583	+	ENSG00000223850	MYCNUT
		rs77702691		−1	−1			
		rs9389266	6	135090599	135090599	+	ENSG00000112339	HBS1L
		rs9567264	13	32146619	32146619	+	ENSG00000073910	FRY
	Lachnospira	rs13157098		−1	−1			
		rs159484	4	111074471	111074471	+	ENSG00000288692	ENSG00000288692
		rs2520509	12	90612737	90612737	+	ENSG00000286021	LINC02822
		rs4686798	3	186727647	186727647	+	ENSG00000113889	KNG1
		rs4923324		−1	−1			
		rs56791201		−1	−1			
	Eubacterium	rs12129908		−1	−1			
		rs12423772		−1	−1			
		rs1425962	4	186984245	186984245	+	ENSG00000249742	ENSG00000249742
		rs2973294	4	37525057	37525057	+	ENSG00000154274	C4orf19
		rs34561138		−1	−1			
	Veillonella	rs1882878	21	28638351	28638351	+	ENSG00000232855	ENSG00000232855
		rs2013594	11	44280604	44280604	+	ENSG00000052850	ALX4
		rs55807413		−1	-1			
		rs62376424	5	120578590	120578590	+	ENSG00000184838	PRR16
		rs6656807		-1	-1			
		rs7359080	14	106483909	106483909	+	ENSG00000270816	LINC00221
		rs742016	22	45208919	45208919	+	ENSG00000100364	KIAA0930

**Figure 4:**
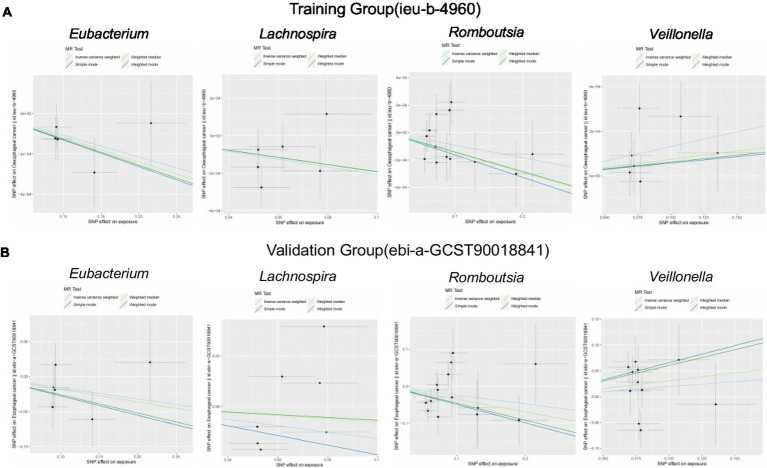
Scatterplot of correlation between training and validation groups. **(A)** Scatterplot of the causal relationship between the four intestinal flora and ESCA in the training group. **(B)** Scatterplot of the causal relationship between the four intestinal flora and ESCA in the validation group.

**Figure 5:**
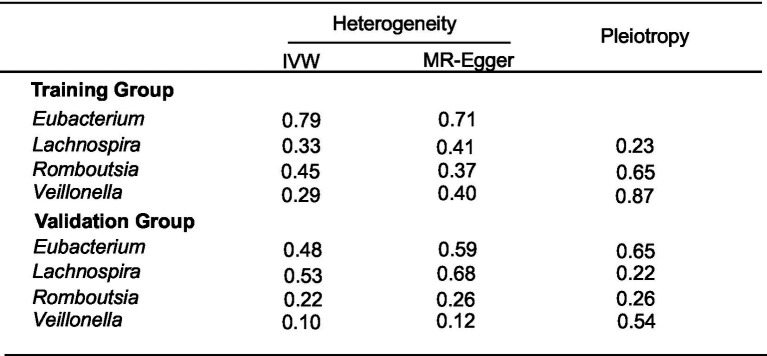
Heterogeneity and pleiotropy results of training and validation group analyses.

We brought the above four intestinal flora into the external dataset for further analysis, and by plotting scatter plots for the training group (Figure 4A) and the validation group (Figure 4B), we found that the four intestinal flora in the validation group had the same tendency to have the same effect on ESCA when compared with the experimental group, which further validated our results. Meanwhile, we further counted the heterozygosity and pleiotropy of the training and validation group analyses (Figure 5), which showed that none of them had pleiotropy, indicating the credibility of our results.

### Meta analysis

**Figure 6:**
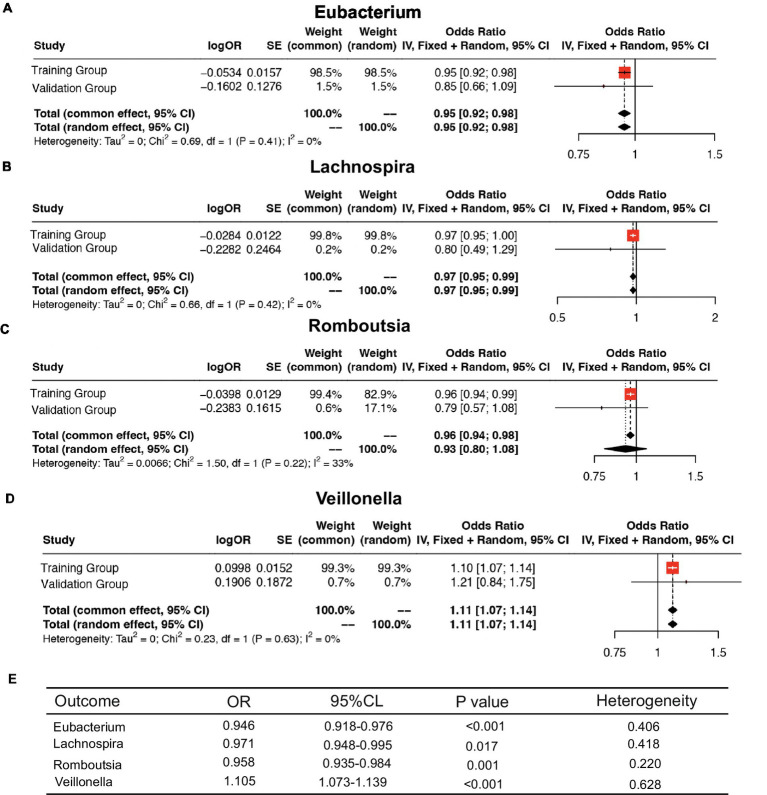
Meta-analysis of the causal association between host gene–gut microbiota and ESCA. **(A–D)** Meta-analysis of the causal relationship between four intestinal flora and ESCA. **(E)** Meta-analysis results.

We further meta-analyzed and plotted and mapped the correlation data of the training and validation groups with the four intestinal flora in a forest plot (Figures 6A–D), which showed that the above relationship was robust. For ease of analysis, we summarized the results into a three-line table (Figure 6E), which showed less statistical heterogeneity and statistically significant results, which further justified our conclusions above.

## Discussion

The esophagus in the gastrointestinal tract is colonized by various microorganisms and is not sterile (Laserna-Mendieta et al., 2021). Healthy individuals have a relatively stable pH (approximately 7) that provides a stable environment for microbial survival (Hasan et al., 2021). The gut microbiota can directly or indirectly affect human health and disease and is considered a new “organ” (Baquero and Nombela, 2012). Gut microbes significantly impact cell formation, differentiation, metabolism, and growth. Dysbiotic gut microbiota may contribute to the body’s carcinogenic process (Zhou et al., 2021; Gou et al., 2023; Guevara-Ramirez et al., 2023). Notably, the gut microbiota may be involved in the development of ESCA (Lv et al., 2019; Zhou et al., 2021). For example, human papillomavirus (HPV) and alterations in intestinal bacteria may cause ESCA (Meng et al., 2018). Moreover, in regions with a high incidence of ESCA, a high prevalence of HPV has often been reported (Yano et al., 2021). Deng et al. studied 23 patients with ESCA and 23 healthy individuals and observed that patients with ESCA have higher levels of Firmicutes and Actinobacteria and lower levels of Bacteroidetes (Deng et al., 2021). This suggests that the gut microbiota and development of ESCA are closely related. In addition, changes in the gut microbiota can increase the levels of pro-inflammatory cytokines and immune cells, thereby inducing tumorigenesis (Proano-Vasco et al., 2021). Notably, gut microbiota can induce the overexpression of nitric oxide synthase, potentially leading to ESCA (Gillespie et al., 2021). The gut microbiota can also interact with the host by secreting bioactive substances, such as vitamins (Malesza et al., 2021), that can be beneficial or harmful for the organism (Riwes and Reddy, 2018). These explanations help to clarify how the gut microbiota and ESCA are related.

In this study, MR analysis was used to assess the causal relationship between gut microbiota and ESCA for providing a theoretical foundation for the interactions between ESCA and gut microbiota, given the lack of conclusive evidence to support the potential relationship between gut microbiota and ESCA. The results of this study showed that four intestinal microbiota were potentially associated with ESCA. Specifically, the genera *Romboutsia*, *Lachnospira*, and *Eubacterium* were negatively associated with ESCA, whereas *Veillonella* had a positive causal relationship with ESCA.

The genus *Romboutsia* is a group of gram-positive bacteria first proposed in 2014 by Ricaboni et al. (2016) from the right half of the human colon using colonoscopy. Most studies concluded that *Romboutsia* belongs to the natural gut microbial community and plays an essential role in host health. For example, *Lactobacillus acidophilus* ameliorates colitis by increasing the abundance of *Romboutsia* (Han et al., 2023). Several studies have found that *Romboutsia* is significantly less abundant in patients with inflammatory bowel disease (e.g., Crohn’s disease, ulcerative colitis) (Qiu et al., 2020; Wang et al., 2023), and is associated with hepatocellular liver cancer and postherpetic neuralgia (Feng et al., 2023; Jiao et al., 2023). Consequently, *Romboutsia* may influence immune regulation and intestinal health (Liu et al., 2023). At the same time *Romboutsia* is able to increase the antioxidant capacity of the body (Zhang et al., 2023), attenuates intestinal inflammatory damage and inhibits endoplasmic reticulum stress, which may be the mechanism by which it affects human health (Li et al., 2023). Moreover, *Romboutsia* is closely associated with esophageal epithelial atrophy (Pan et al., 2021), an essential stage in the development of ESCA. The potential relationship between *Romboutsia* and ESCA identified in this study is consistent with the above findings, which suggests that our analysis is logical.

*Lachnospira* is integral to the gut microbiota and colonizes the intestinal lumen from birth (Vacca et al., 2020). It is a group of potentially beneficial bacteria involved in the metabolism of various carbohydrates. Fermentation produces acetic and butyric acids, which provide energy to the host (Devillard et al., 2007; Wong et al., 2014). *Lachnospira* has been implicated in various diseases, including obesity (Natividad et al., 2018), liver disease (De Minicis et al., 2014), and chronic kidney disease (Yasuno et al., 2023), and can also lead to depression via the gut–brain axis (Bonaz et al., 2018). The *Lachnospira* spp. have anti-inflammatory and antioxidant effects and can protect the intestinal mucosal barrier by inhibiting inflammatory responses and scavenging free radicals, which may be a mechanism for preventing esophageal diseases (Mukherjee et al., 2020). A cohort study showed that *Lachnospira*, which has anti-inflammatory properties, was significantly reduced in ESCA patients compared to the normal group (Cheung et al., 2022). This is consistent with our findings in the current study.

*Eubacterium* has been found to be associated with age-related macular degeneration (AMD) (Mao et al., 2023), female infertility (Xi et al., 2023), multiple sclerosis (MS) (Vacaras et al., 2023) and other diseases. Although a previous Mendelian analysis done by Yang et al. showed that *Eubacterium* reduced the risk of Barrett’s esophagus (Yang et al., 2022), the literature exploring the relationship between *Eubacterium* spp. and ESCA remains scarce to date. This is the innovative finding of our present study, and it also suggests an interesting research direction for us. Now, our experiments on *Eubacterium* for the prevention of ESCA are in progress, and the results will be published in a follow-up study.

The genus *Veillonella* includes gram-negative, anaerobic, non-motile, and non-spore-forming coccus bacteria (Djais et al., 2019). *Veillonella* is strongly associated with the development of several diseases, and has been found to promote the proliferation of lung adenocarcinoma (Zeng et al., 2023), whilst *Veillonella* activates macrophages to promote inflammatory responses via the LPS-TLR4 pathway (Zhan et al., 2022), for example, *Veillonella* correlated with the severity of radiation esophagitis (Lin et al., 2022), and inflammation is one of the factors leading to esophageal cancer, suggesting that *Veillonella* may indirectly contribute to esophageal carcinogenesis through inflammation. In our study, only the genus *Veillonella* positively correlated with ESCA. An extensive body of literature describes the pathogenic role of *Veillonella* in the esophagus. For example, a study using whole genome sequencing (wGS) and RNA sequencing (rNAseq) of tumors from 61 patients with ESCA found a high abundance of *Veillonella* in ESCA (Nomburg et al., 2022). It has also been shown that *Veillonella* levels gradually increase during the development of esophageal reflux (GR)-Barrett’s esophagus (BE)-esophageal adenocarcinoma (EA) (Di Pilato et al., 2016; Hao et al., 2022). Gram-negative bacteria are mainly associated with esophageal abnormalities, e.g., *Veillonella* is mostly associated with BE (Lv et al., 2019; Park and Lee, 2020), which aligns with our findings. These findings have important implications for research in ESCA in the future and may inspire new prevention and treatment strategies for this disease.

Many previous studies have demonstrated a potential relationship between the gut microbiota and ESCA (Baba et al., 2023; Co et al., 2023; Sugimoto et al., 2023). However, most of these studies were observational and susceptible to confounding factors. In contrast, the present study used a genetic epidemiological approach to minimize the influence of confounding factors and provide a compelling insight into the relationship between gut microbiota and ESCA. And the use of external datasets to validate trends and Meta-analysis of the results ensures maximum stability of the results.

Gut flora plays a dual role in cancer development, and the use of gut flora in conjunction with traditional antitumor treatment strategies, as well as the use of probiotics, FMT, and dietary control, can improve the efficacy of anticancer treatments, while reducing the incidence of side effects and improving prognosis (Sun et al., 2023). However, this study had certain limitations. First, most of the participants were European. Additional research is required to determine whether these conclusions apply to other ethnic groups. Second, the flora in this study was limited to the genus level and was not further subdivided.

## Conclusion

We analyzed the potential relationship between 196 common intestinal microbiota and ESCA. We found that the genera *Romboutsia*, *Lachnospira*, *Eubacterium*, and *Veillonella* may be causally associated with ESCA, which may provide new ideas for ESCA research and treatment.

## Data availability statement

The original contributions presented in the study are included in the article/Supplementary material, further inquiries can be directed to the corresponding author.

## Ethics statement

Ethical approval was not required for this study, because data were obtained from publicly available databases and no identifiable data was published.

## Author contributions

ZZ: Writing – original draft. GZ: Conceptualization, Writing – review & editing. ZH: Methodology, Writing – review & editing. YS: Investigation, Writing – review & editing. DW: Writing – original draft.

## Footnotes

^1^ https://gwas.mrcieu.ac.uk/

^2^ http://www.phenoscanner.medschl.cam.ac.uk/

^3^ https://biit.cs.ut.ee/gprofiler/snpense

## Supplementary material

### SUPPLEMENTARY FIGURE 1

Leave-one-out analysis forest plot.

### SUPPLEMENTARY TABLE 1

13 SNPs associated with ESCA in *Romboutsia*.

### SUPPLEMENTARY TABLE 2

6 SNPs associated with ESCA in *Lachnospira*.

### SUPPLEMENTARY TABLE 3

5 SNPs associated with ESCA in *Eubacterium*.

### SUPPLEMENTARY TABLE 4

7 SNPs associated with ESCA in *Veillonella*.

## References

[ref1] Baba Y. Hara Y. Toihata T. Kosumi K. Iwatsuki M. Iwagami S.. (2023). Relationship between gut microbiome fusobacterium nucleatum and LINE−1 methylation level in esophageal cancer. Esophagus 20, 704–712. doi: 10.1007/s10388-023-01009-9, PMID: 37173453

[ref2] Baquero F. Nombela C. (2012). The microbiome as a human organ. Clin. Microbiol. Infect. 18, 2–4. doi: 10.1111/j.1469-0691.2012.03916.x 22647038

[ref3] Bonaz B. Bazin T. Pellissier S. (2018). The Vagus nerve at the Interface of the microbiota-gut-brain Axis. Front. Neurosci. 12:49. doi: 10.3389/fnins.2018.00049, PMID: 29467611 PMC5808284

[ref4] Burgess S. Thompson S. G. (2017). Interpreting findings from Mendelian randomization using the MR-egger method. Eur. J. Epidemiol. 32, 377–389. doi: 10.1007/s10654-017-0255-x, PMID: 28527048 PMC5506233

[ref5] Cheung M. K. Yue G. G. L. Lauw S. Li C. S. Y. Yung M. Y. Ng S. C.. (2022). Alterations in gut microbiota of esophageal squamous cell carcinoma patients. J. Gastroenterol. Hepatol. 37, 1919–1927. doi: 10.1111/jgh.15941, PMID: 35816164

[ref6] Co E. L. Hameed M. Sebastian S. A. Garg T. Sudan S. Bheemisetty N.. (2023). Narrative review of probiotic use on the recovery of postoperative patients with esophageal Cancer. Curr. Nutr. Rep. doi: 10.1007/s13668-023-00490-z, PMID: 37605086

[ref7] De Minicis S. Rychlicki C. Agostinelli L. Saccomanno S. Candelaresi C. Trozzi L.. (2014). Dysbiosis contributes to fibrogenesis in the course of chronic liver injury in mice. Hepatology 59, 1738–1749. doi: 10.1002/hep.26695 23959503

[ref8] Deng Y. Tang D. Hou P. Shen W. Li H. Wang T.. (2021). Dysbiosis of gut microbiota in patients with esophageal cancer. Microb. Pathog. 150:104709. doi: 10.1016/j.micpath.2020.104709, PMID: 33378710

[ref9] Devillard E. McIntosh F. M. Duncan S. H. Wallace R. J. (2007). Metabolism of linoleic acid by human gut bacteria: different routes for biosynthesis of conjugated linoleic acid. J. Bacteriol. 189, 2566–2570. doi: 10.1128/JB.01359-06, PMID: 17209019 PMC1899373

[ref10] Di Pilato V. Freschi G. Ringressi M. N. Pallecchi L. Rossolini G. M. Bechi P. (2016). The esophageal microbiota in health and disease. Ann. N. Y. Acad. Sci. 1381, 21–33. doi: 10.1111/nyas.13127, PMID: 27415419

[ref11] Djais A. A. Theodorea C. F. Mashima I. Otomo M. Saitoh M. Nakazawa F. (2019). Identification and phylogenetic analysis of oral Veillonella species isolated from the saliva of Japanese children. F1000Res 8:616. doi: 10.12688/f1000research.18506.5, PMID: 31448103 PMC6688723

[ref12] Feng J. Wu Y. Dai P. Wang D. Liu L. Chai B. (2023). Gut microbial signatures of patients with primary hepatocellular carcinoma and their healthy first-degree relatives. J. Appl. Microbiol. 134:221. doi: 10.1093/jambio/lxad221, PMID: 37777841

[ref13] Gillespie M. R. Rai V. Agrawal S. Nandipati K. C. (2021). The role of microbiota in the pathogenesis of esophageal adenocarcinoma. Biology (Basel) 10:697. doi: 10.3390/biology10080697 34439930 PMC8389269

[ref14] Gou H. Su H. Liu D. Wong C. C. Shang H. Fang Y.. (2023). Traditional medicine Pien Tze Huang suppresses colorectal tumorigenesis through restoring gut microbiota and metabolites. Gastroenterology 165, 1404–1419. doi: 10.1053/j.gastro.2023.08.052, PMID: 37704113

[ref15] Guevara-Ramirez P. Cadena-Ullauri S. Paz-Cruz E. Tamayo-Trujillo R. Ruiz-Pozo V. A. Zambrano A. K. (2023). Role of the gut microbiota in hematologic cancer. Front. Microbiol. 14:1185787. doi: 10.3389/fmicb.2023.1185787, PMID: 37692399 PMC10485363

[ref16] Han M. Liao W. Dong Y. Fei T. Gai Z. (2023). Sustained ameliorative effect of *Lactobacillus acidophilus* LA85 on dextran sulfate sodium-induced colitis in mice. J. Food Sci. 88, 3893–3904. doi: 10.1111/1750-3841.16723, PMID: 37548631

[ref17] Hao Y. Karaoz U. Yang L. Yachimski P. S. Tseng W. Nossa C. W.. (2022). Progressive dysbiosis of human orodigestive microbiota along the sequence of gastroesophageal reflux, Barrett's esophagus and esophageal adenocarcinoma. Int. J. Cancer 151, 1703–1716. doi: 10.1002/ijc.34191, PMID: 35751398

[ref18] Hasan A. Hasan L. K. Schnabl B. Greytak M. Yadlapati R. (2021). Microbiome of the Aerodigestive tract in health and esophageal disease. Dig. Dis. Sci. 66, 12–18. doi: 10.1007/s10620-020-06720-6, PMID: 33236315 PMC8006547

[ref19] He S. Xu J. Liu X. Zhen Y. (2021). Advances and challenges in the treatment of esophageal cancer. Acta Pharm. Sin. B 11, 3379–3392. doi: 10.1016/j.apsb.2021.03.008, PMID: 34900524 PMC8642427

[ref20] Jiao B. Cao X. Zhang C. Zhang W. Yu S. Zhang M.. (2023). Alterations of the gut microbiota in patients with postherpetic neuralgia. AMB Express 13:108. doi: 10.1186/s13568-023-01614-y, PMID: 37803181 PMC10558420

[ref21] Kurilshikov A. Medina-Gomez C. Bacigalupe R. Radjabzadeh D. Wang J. Demirkan A.. (2021). Large-scale association analyses identify host factors influencing human gut microbiome composition. Nat. Genet. 53, 156–165. doi: 10.1038/s41588-020-00763-1, PMID: 33462485 PMC8515199

[ref22] Laserna-Mendieta E. J. FitzGerald J. A. Arias-Gonzalez L. Ollala J. M. Bernardo D. Claesson M. J.. (2021). Esophageal microbiome in active eosinophilic esophagitis and changes induced by different therapies. Sci. Rep. 11:7113. doi: 10.1038/s41598-021-86464-z, PMID: 33782490 PMC8007638

[ref23] Li N. Bai C. Zhao L. Ge Y. Li X. (2022). Characterization of the fecal microbiota in gastrointestinal cancer patients and healthy people. Clin. Transl. Oncol. 24, 1134–1147. doi: 10.1007/s12094-021-02754-y, PMID: 35167015

[ref24] Li K. Wu J. Xu S. Li X. Zhang Y. Gao X. J. (2023). Rosmarinic acid alleviates intestinal inflammatory damage and inhibits endoplasmic reticulum stress and smooth muscle contraction abnormalities in intestinal tissues by regulating gut microbiota. Microbiol. Spectr. 11:1914. doi: 10.1128/spectrum.01914-23 PMC10654191 37594285

[ref25] Lin M. Q. Wu Y. H. Yang J. Lin H. C. Liu L. Y. Yu Y. L.. (2022). Gut microbiota characteristics are associated with severity of acute radiation-induced esophagitis. Front. Microbiol. 13:883650. doi: 10.3389/fmicb.2022.883650, PMID: 35756007 PMC9218355

[ref26] Liu J. Cai J. Fan P. Dong X. Zhang N. Tai J.. (2023). Salidroside alleviates dextran sulfate sodium-induced colitis in mice by modulating the gut microbiota. Food Funct. 14, 7506–7519. doi: 10.1039/D3FO01929B, PMID: 37504971

[ref27] Lv J. Guo L. Liu J. J. Zhao H. P. Zhang J. Wang J. H. (2019). Alteration of the esophageal microbiota in Barrett's esophagus and esophageal adenocarcinoma. World J. Gastroenterol. 25, 2149–2161. doi: 10.3748/wjg.v25.i18.2149, PMID: 31143067 PMC6526156

[ref28] Malesza I. J. Malesza M. Walkowiak J. Mussin N. Walkowiak D. Aringazina R.. (2021). High-Fat, western-style diet, systemic inflammation, and gut microbiota: A narrative review. Cells 10:3164. doi: 10.3390/cells10113164 34831387 PMC8619527

[ref29] Mao D. Tao B. Sheng S. Jin H. Chen W. Gao H.. (2023). Causal effects of gut microbiota on age-related macular degeneration: a Mendelian randomization study. Invest. Ophthalmol. Vis. Sci. 64:32. doi: 10.1167/iovs.64.12.32, PMID: 37725382 PMC10513115

[ref30] Meng C. Bai C. Brown T. D. Hood L. E. Tian Q. (2018). Human gut microbiota and gastrointestinal Cancer. Genomics Proteomics Bioinformatics 16, 33–49. doi: 10.1016/j.gpb.2017.06.002, PMID: 29474889 PMC6000254

[ref31] Mukherjee A. Lordan C. Ross R. P. Cotter P. D. (2020). Gut microbes from the phylogenetically diverse genus Eubacterium and their various contributions to gut health. Gut Microbes 12:1802866. doi: 10.1080/19490976.2020.1802866, PMID: 32835590 PMC7524325

[ref32] Muszynski D. Kudra A. Sobocki B. K. Folwarski M. Vitale E. Filetti V.. (2022). Esophageal cancer and bacterial part of gut microbiota – a multidisciplinary point of view. Front. Cell. Infect. Microbiol. 12:1057668. doi: 10.3389/fcimb.2022.1057668, PMID: 36467733 PMC9709273

[ref33] Natividad J. M. Agus A. Planchais J. Lamas B. Jarry A. C. Martin R.. (2018). Impaired aryl hydrocarbon receptor ligand production by the gut microbiota is a key factor in metabolic syndrome. Cell Metab. 28:e734, 737–749.e4. doi: 10.1016/j.cmet.2018.07.001 30057068

[ref34] Nomburg J. Bullman S. Nasrollahzadeh D. Collisson E. A. Abedi-Ardekani B. Akoko L. O.. (2022). An international report on bacterial communities in esophageal squamous cell carcinoma. Int. J. Cancer 151, 1947–1959. doi: 10.1002/ijc.34212, PMID: 35837755 PMC11100422

[ref35] Ohkusa T. Nishikawa Y. Sato N. (2022). Gastrointestinal disorders and intestinal bacteria: advances in research and applications in therapy. Front. Med. 9:935676. doi: 10.3389/fmed.2022.935676 PMC9941163 36825261

[ref36] Oppedijk V. van der Gaast A. van Lanschot J. J. van Hagen P. van Os R. van Rij C. M.. (2014). Patterns of recurrence after surgery alone versus preoperative chemoradiotherapy and surgery in the CROSS trials. J. Clin. Oncol. 32, 385–391. doi: 10.1200/JCO.2013.51.2186, PMID: 24419108

[ref37] Pan F. Zhang L. L. Luo H. J. Chen Y. Long L. Wang X.. (2021). Dietary riboflavin deficiency induces ariboflavinosis and esophageal epithelial atrophy in association with modification of gut microbiota in rats. Eur. J. Nutr. 60, 807–820. doi: 10.1007/s00394-020-02283-4, PMID: 32458157

[ref38] Park C. H. Lee S. K. (2020). Exploring esophageal microbiomes in esophageal diseases: a systematic review. J. Neurogastroenterol. Motil. 26, 171–179. doi: 10.5056/jnm19240, PMID: 32235026 PMC7176507

[ref39] Proano-Vasco A. Baumeister T. Metwaly A. Reitmeier S. Kleigrewe K. Meng C.. (2021). High-fructose diet alters intestinal microbial profile and correlates with early tumorigenesis in a mouse model of Barrett's esophagus. Microorganisms 9:2432. doi: 10.3390/microorganisms9122432, PMID: 34946037 PMC8708753

[ref40] Qiu X. Zhao X. Cui X. Mao X. Tang N. Jiao C.. (2020). Characterization of fungal and bacterial dysbiosis in young adult Chinese patients with Crohn's disease. Ther. Adv. Gastroenterol. 13:175628482097120. doi: 10.1177/1756284820971202 PMC7672770 33240394

[ref41] Ricaboni D. Mailhe M. Khelaifia S. Raoult D. Million M. (2016). Romboutsia timonensis, a new species isolated from human gut. New Microbes New Infect. 12, 6–7. doi: 10.1016/j.nmni.2016.04.001, PMID: 27200178 PMC4864248

[ref42] Riwes M. Reddy P. (2018). Microbial metabolites and graft versus host disease. Am. J. Transplant. 18, 23–29. doi: 10.1111/ajt.14443 28742948

[ref43] Smith G. D. Ebrahim S. (2003). 'Mendelian randomization': can genetic epidemiology contribute to understanding environmental determinants of disease? Int. J. Epidemiol. 32, 1–22. doi: 10.1093/ije/dyg070, PMID: 12689998

[ref44] Sugimoto T. Atobe S. Kado Y. Takahashi A. Motoori M. Sugimura K.. (2023). Gut microbiota associated with the mitigation effect of synbiotics on adverse events of neoadjuvant chemotherapy in patients with esophageal cancer: a retrospective exploratory study. J. Med. Microbiol. 72:1723. doi: 10.1099/jmm.0.001723, PMID: 37367942

[ref45] Sun J. Chen F. Wu G. (2023). Potential effects of gut microbiota on host cancers: focus on immunity, DNA damage, cellular pathways, and anticancer therapy. ISME J. 17, 1535–1551. doi: 10.1038/s41396-023-01483-0 37553473 PMC10504269

[ref46] Sung H. Ferlay J. Siegel R. L. Laversanne M. Soerjomataram I. Jemal A.. (2021). Global Cancer statistics 2020: GLOBOCAN estimates of incidence and mortality worldwide for 36 cancers in 185 countries. CA Cancer J. Clin. 71, 209–249. doi: 10.3322/caac.21660, PMID: 33538338

[ref47] Thanawala S. U. Kaplan D. E. Falk G. W. Beveridge C. A. Schaubel D. Serper M.. (2023). Antibiotic exposure is associated with a risk of esophageal adenocarcinoma. Clin. Gastroenterol. Hepatol. 21, 2817–2824.e4. doi: 10.1016/j.cgh.2023.03.012, PMID: 36967101 PMC10518027

[ref48] Uhlenhopp D. J. Then E. O. Sunkara T. Gaduputi V. (2020). Epidemiology of esophageal cancer: update in global trends, etiology and risk factors. Clin. J. Gastroenterol. 13, 1010–1021. doi: 10.1007/s12328-020-01237-x, PMID: 32965635

[ref49] Vacaras V. Muresanu D. F. Buzoianu A. D. Nistor C. Vesa S. C. Paraschiv A. C.. (2023). The role of multiple sclerosis therapies on the dynamic of human gut microbiota. J. Neuroimmunol. 378:578087. doi: 10.1016/j.jneuroim.2023.578087, PMID: 37058852

[ref50] Vacca M. Celano G. Calabrese F. M. Portincasa P. Gobbetti M. De Angelis M. (2020). The controversial role of human gut Lachnospiraceae. Microorganisms 8:573. doi: 10.3390/microorganisms8040573, PMID: 32326636 PMC7232163

[ref51] Verbanck M. Chen C. Y. Neale B. Do R. (2018). Publisher correction: detection of widespread horizontal pleiotropy in causal relationships inferred from Mendelian randomization between complex traits and diseases. Nat. Genet. 50:1196. doi: 10.1038/s41588-018-0164-2, PMID: 29967445

[ref52] Wang K. Qin L. Cao J. Zhang L. Liu M. Qu C.. (2023). Kappa-Selenocarrageenan oligosaccharides prepared by Deep-Sea enzyme alleviate inflammatory responses and modulate gut microbiota in ulcerative colitis mice. Int. J. Mol. Sci. 24:4672. doi: 10.3390/ijms24054672 36902109 PMC10003262

[ref53] Wong J. Piceno Y. M. DeSantis T. Z. Pahl M. Andersen G. L. Vaziri N. D. (2014). Expansion of urease-and uricase-containing, indole-and p-cresol-forming and contraction of short-chain fatty acid-producing intestinal microbiota in ESRD. Am. J. Nephrol. 39, 230–237. doi: 10.1159/000360010, PMID: 24643131 PMC4049264

[ref54] Xi Y. Zhang C. Feng Y. Zhao S. Zhang Y. Duan G.. (2023). Genetically predicted the causal relationship between gut microbiota and infertility: bidirectional Mendelian randomization analysis in the framework of predictive, preventive, and personalized medicine. EPMA J. 14, 405–416. doi: 10.1007/s13167-023-00332-6, PMID: 37605651 PMC10439866

[ref55] Yang Z. Yu R. Deng W. Wang W. (2022). Roles of 21 genera of human gut microbiota in Barrett's esophagus risk: a Mendelian randomization study. Front. Genet. 13:894900. doi: 10.3389/fgene.2022.894900, PMID: 35754845 PMC9219910

[ref56] Yano Y. Etemadi A. Abnet C. C. (2021). Microbiome and cancers of the esophagus: A Review. Microorganisms 9:764. doi: 10.3390/microorganisms9081764 34442842 PMC8398938

[ref57] Yasuno T. Takahashi K. Tada K. Hiyamuta H. Watanabe M. Ito K.. (2023). Dysbiosis of gut microbiota in patients with chronic kidney disease. Intern. Med. doi: 10.2169/internalmedicine.1602-23 PMC10901723 37344438

[ref58] Zeng W. Wang Y. Wang Z. Yu M. Liu K. Zhao C.. (2023). *Veillonella parvula* promotes the proliferation of lung adenocarcinoma through the nucleotide oligomerization domain 2/cellular communication network factor 4/nuclear factor kappa B pathway. Discov. Oncol. 14:129. doi: 10.1007/s12672-023-00748-6, PMID: 37452162 PMC10349017

[ref59] Zhan Z. Liu W. Pan L. Bao Y. Yan Z. Hong L. (2022). Overabundance of *Veillonella parvula* promotes intestinal inflammation by activating macrophages via LPS-TLR4 pathway. Cell Death Discov. 8:251. doi: 10.1038/s41420-022-01015-3, PMID: 35523778 PMC9076897

[ref60] Zhang P. Jiang G. Wang Y. Yan E. He L. Guo J.. (2023). Maternal consumption of l-malic acid enriched diets improves antioxidant capacity and glucose metabolism in offspring by regulating the gut microbiota. Redox Biol. 67:102889. doi: 10.1016/j.redox.2023.102889, PMID: 37741046 PMC10519833

[ref61] Zhou J. Sun S. Luan S. Xiao X. Yang Y. Mao C.. (2021). Gut microbiota for esophageal Cancer: role in carcinogenesis and clinical implications. Front. Oncol. 11:717242. doi: 10.3389/fonc.2021.717242, PMID: 34733778 PMC8558403

