# Supplementary figures and images for: Application of Mendelian randomization to assess host gene–gut microbiota correlations in patients with esophageal cancer
Source: Front Microbiol. 2023 Dec 21;14:1309596. doi: 10.3389/fmicb.2023.1309596 (PMC10764629; doi:10.3389/fmicb.2023.1309596)

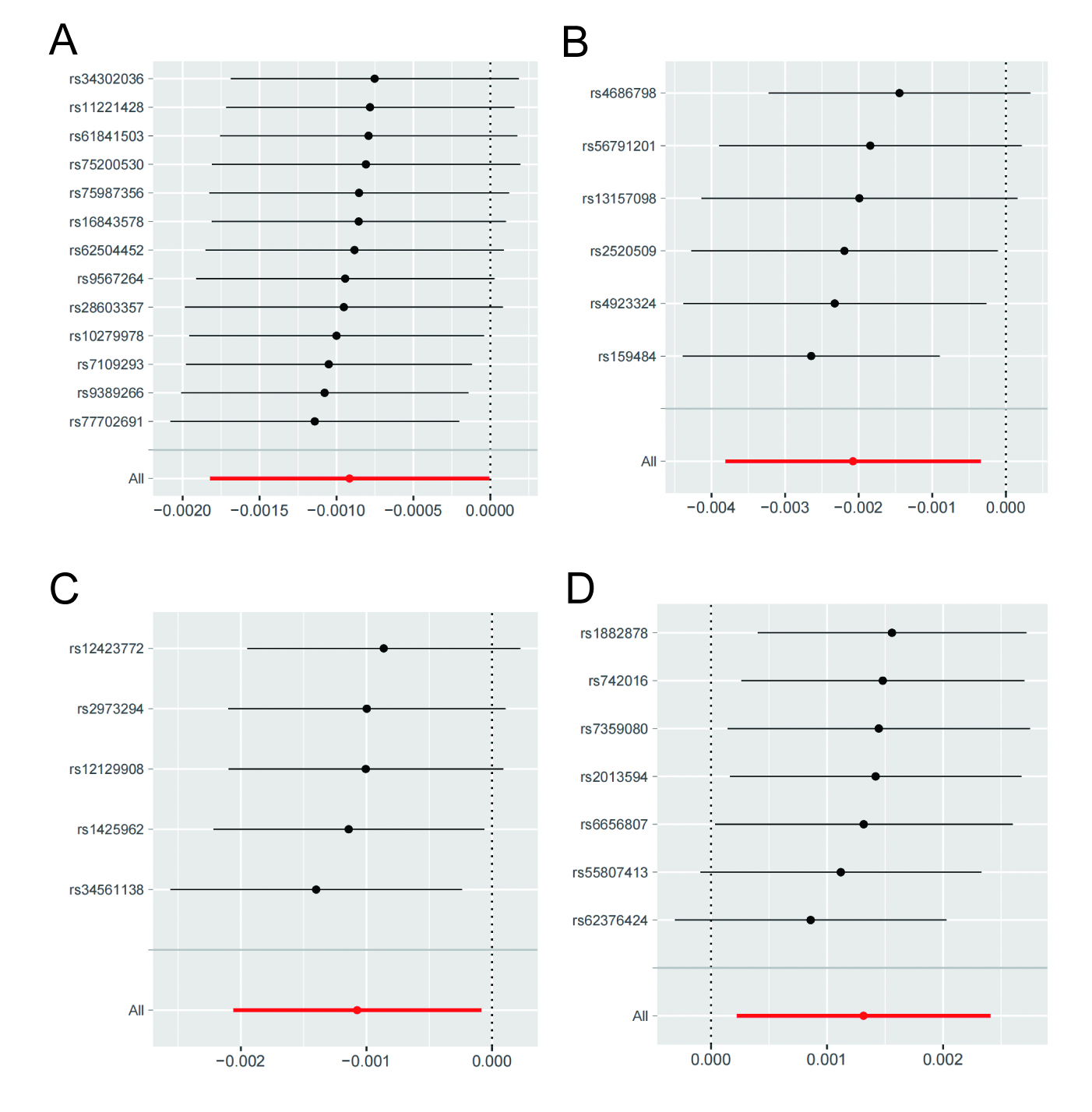

Supplement: Supplementary file 1 [file Data_Sheet_1.zip › Supplementary materials/Figure S1.tif]
